# Effect of a Smartphone-Based Neonatal Transitional Care Program for Families of Infants and Toddlers Born as High-Risk Neonates

**DOI:** 10.3390/healthcare14193349

**Published:** 2026-10-08

**Authors:** Jeong Soon Kim, Yoon Kyung Kim

**Affiliations:** 1 Department of Nursing, Chungwoon University, Hongseong 32244, Republic of Korea; jskim2022@chungwoon.ac.kr; 2 School of Nursing, Cheju Halla University, Jeju 63092, Republic of Korea

**Keywords:** high-risk neonate, family, transitional care, family-centered care

## Abstract

**Highlights:**

**What are the main findings?**

- A smartphone-based NTC (“Developing Together”) program increased parental knowledge and confidence while lowering parenting stress in families of infants and toddlers born as high-risk neonates.

**What are the implications of the main findings?**

- Establishing community partnerships through mobile NTCs enables the provision of equitable transitional care even in small towns—areas with limited access to services—and supports sustainable operations.
- In NTC programs for families, adding peer support from program graduates, structured expert coaching, and interactive modules can further strengthen parents’ parenting skills and emotional support.

**Abstract:**

**Background:** In this study, researchers developed and evaluated the effect of a neonatal transitional care program application designed for home use by families of infants and toddlers born as high-risk neonates following discharge from the neonatal intensive care unit. **Methods:** The and “Developing Together” (neonatal transitional care, NTC) program, a smartphone-based application, was created based on the FICare model, utilizing the Analysis, Design, Develop, Implement, and Evaluate instructional design process. Researchers employed a nonequivalent control group and non-synchronized design (experimental group, *n* = 65; control group, *n* = 63). The program was conducted over 5–6 weeks. Participants were evaluated using questionnaires before and immediately after the program as an online, self-administered questionnaire. Data were analyzed using descriptive statistics, *t*-tests, χ^2^ tests and Fisher’s exact tests, in addition to ANCOVA. **Results:** Compared with the parents in the control group, those in the experimental group demonstrated increased developmental knowledge (F = 3.94, *p* = 0.009), parenting knowledge (F = 6.72, *p* < 0.001) and maternal confidence (F = 9.33, *p* < 0.001), and reduced stress levels (F = 2.70, *p* = 0.046). **Conclusions:** The transitional care program utilizing a smartphone app led to significant improvements following the educational intervention for families of high-risk neonates within the community.

## 1. Introduction

Globally, preterm births account for approximately 10% of all births [1]. In the Republic of Korea, high-risk newborns comprise 9.9% of births, representing a 50% increase over the past decade [2]. Advances in medical technology have increased preterm infant survival from 84.9% in 2014 to 89.9% in 2022 [2].

Adequate fetal development requires a 40-week gestation, allowing the fetus to attain independent postnatal survival. Preterm birth substantially increases the risk of severe acute illnesses and growth or developmental disorders, affecting both infant development and family quality of life. Families of high-risk neonates face health vulnerabilities, unfamiliar medical environments, disrupted bonding, and increased parenting stress related to developmental uncertainty, negative emotions, and reduced maternal confidence [3,4,5].

Notably, preterm and low-birth-weight neonates without congenital anomalies have prognoses that vary widely according to the quality of postnatal treatment and care. This underscores the importance of neonatal intensive care units (NICUs) and neonatal transitional care (NTC) at home. NTC is a collaborative, family-participatory care system that helps families transition to home life after the acute postnatal period and NICU discharge [6]. Based on the family-centered care (FCC) model, it includes educational and supportive nursing interventions, emotional support, kangaroo care, and therapeutic interventions, positively affecting preterm infant development and family adaptation [4,7,8].

The rapid commercialization of the internet and advances in mobile technology since the early 2010s have reduced time and geographic constraints. Since the coronavirus disease pandemic, these technologies have become essential for medical and social support for individuals requiring isolation. Studies of e-health interventions highlight SMS updates, internet-based telemedicine (e.g., Skype), and online video conferencing as methods for expert counseling and education. Such interventions shorten hospital stays, increase parental satisfaction with their parenting involvement, and reduce stress among families transitioning to home care [9].

Families of high-risk neonates face various vulnerabilities, including infectious disease outbreaks, children’s compromised immunity, and caregiving demands. Smartphone applications can provide effective education through diverse learning and interactive content. Franck et al. [10] reported that smartphone-based parent engagement programs provide valuable information, enhance FCC in the NICU, promote kangaroo care and breastfeeding, and facilitate long-term experience sharing. Collected data can also support collaborative community-based research [10].

Despite global use of this technology in high-risk neonatal care, the Republic of Korea lacks a comprehensive support system. Although preterm births are increasing and neonatal care environments are improving, NTC programs remain limited [7,8]. Financial assistance is available for hospitalization and disability diagnosis, but transitional care remains fragmented [8,11]. Nonprofit organizations occasionally offer NTC education, but programs vary by region and lack sustainability. Parents therefore often rely on nonexpert online communities for mothers for information and support, which may facilitate inaccurate information and commercial exploitation. Developing and expanding programs involving medical and rehabilitation professionals is therefore essential for providing reliable parenting guidance. Smartphone applications could further reduce geographic and temporal barriers to service access and enable continuous, systematic NTC education.

This study introduces the FICare program for families undergoing the transition to home care. The program provides clinical support, parent education, peer support, and record management tailored to their needs. The researchers aimed to adapt the established FICare program for families in the Republic of Korea and develop a parenting guide for high-risk neonates. Ultimately, this study seeks to establish a foundation for implementing NTC in the Republic of Korea.

### Hypotheses

Previous studies have shown that family-centered and digital health interventions can improve parents’ knowledge and reduce parenting stress among parents of high-risk infants [7]. Structured developmental-support education may also increase maternal confidence in postdischarge care [8,9,10]. However, evidence regarding smartphone-based NTC programs at home remains limited. Therefore, we hypothesized that:

**Hypothesis** **1.** *The experimental and control groups receiving the smartphone-based NTC program would differ in developmental knowledge.*

**Hypothesis** **2.** *The experimental and control groups receiving the smartphone-based NTC program would differ in parenting knowledge.*

**Hypothesis** **3.** *The experimental and control groups receiving the smartphone-based NTC program would differ in maternal confidence.*

**Hypothesis** **4.** *The experimental and control groups receiving the smartphone-based NTC program would differ in parenting stress.*

## 2. Theoretical Framework

### Family Integrated Care

This study adopted the Family Integrated Care (FICare) theory, derived from the widely used FCC theory for the care of families of high-risk newborns, as its conceptual framework. FCC emphasizes childcare that addresses both infants and parents but may be limited clinically because NICU nurses and specialists primarily direct care. In contrast, FICare is a medium-range model that positions parents as active partners in infant care. Its key strength is that it assigns parents a central role within the treatment team and promoting collaboration with healthcare professionals during NICU and transitional care [12].

The key components of FICare are as follows: (1) Nurses train and support parents to safely provide NICU care, shifting caregiving responsibilities to parents; (2) Parents have unrestricted visiting hours, spend at least 6 h/day in the NICU, and receive care training; (3) Parents participate in medical rounds, report their infant’s condition, and contribute to clinical decisions; and (4) Trained “alumni” with experience raising high-risk infants support current NICU parents [13] (Table 1).

**Figure 1:**
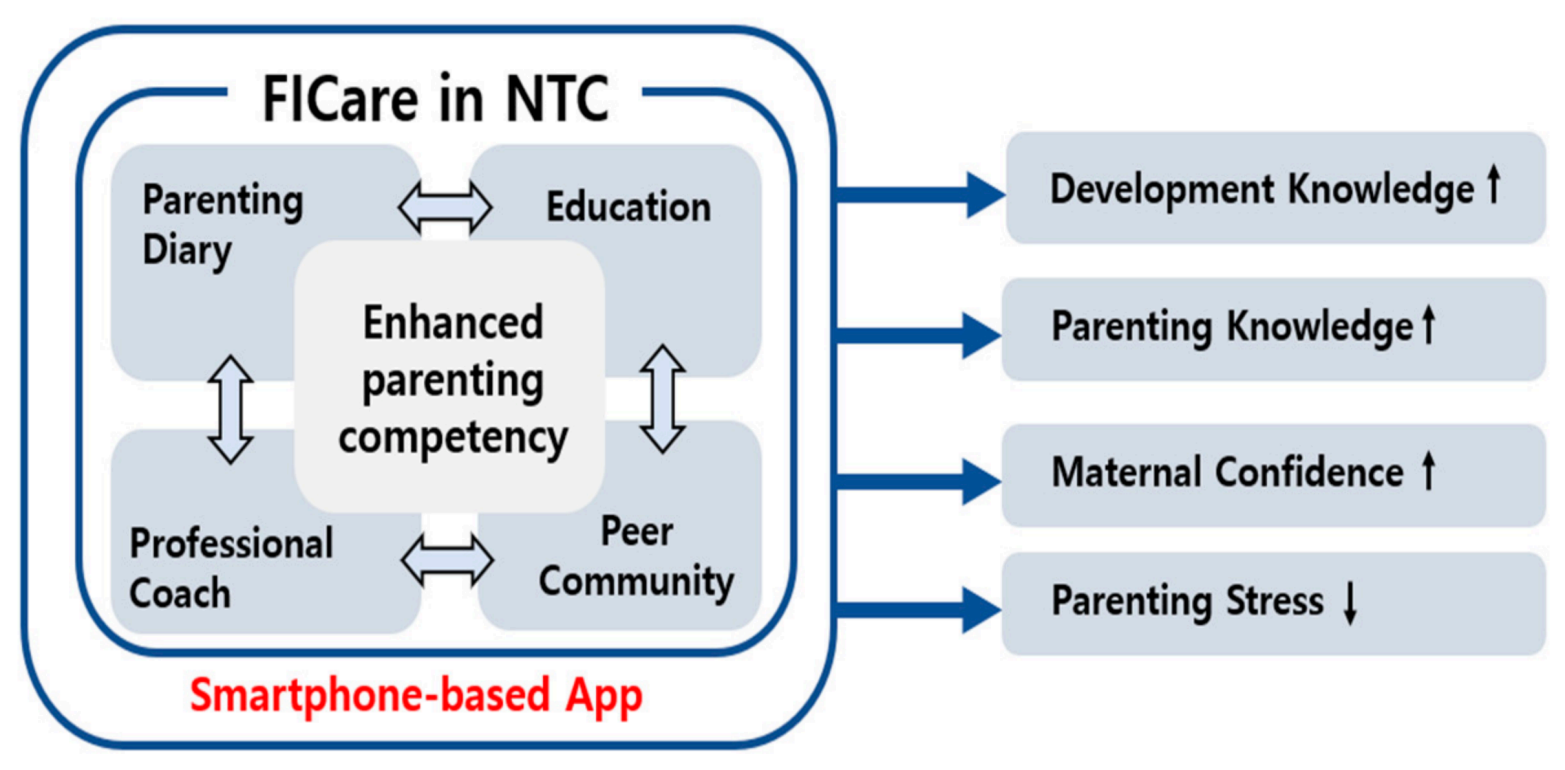
Conceptual framework in study.

A critical element in FICare’s success is strong relationships between NICU staff and parents. The NICU should foster mutual trust by engaging parents as partners in developing the newborn’s treatment plan and care. In contrast, inconsistent communication, passive staff attitudes, and team-centered priorities can hinder FCC and FICare [14,15]. Based on these considerations, the theoretical framework for this study was developed (Figure 1).

## 3. Methods

### 3.1. Study Design

This nonequivalent control group, non-synchronized study evaluated the impact of a smartphone-based NTC program on families of infants and toddlers born as high-risk neonates.

### 3.2. Participants

The target population comprised families caring for infants and toddlers discharged after NICU treatment. Inclusion criteria were as follows: (1) infant age < 36 months; (2) no current acute severe illness; and (3) parents able to use smartphones. Exclusion criteria were as follows: (1) failure to meet the inclusion criteria; (2) parents whose children had emigrated; and (3) parents unable to understand the study information or complete the study procedures.

The accessible population comprised eligible families residing in the Republic of Korea.

**Figure 2:**
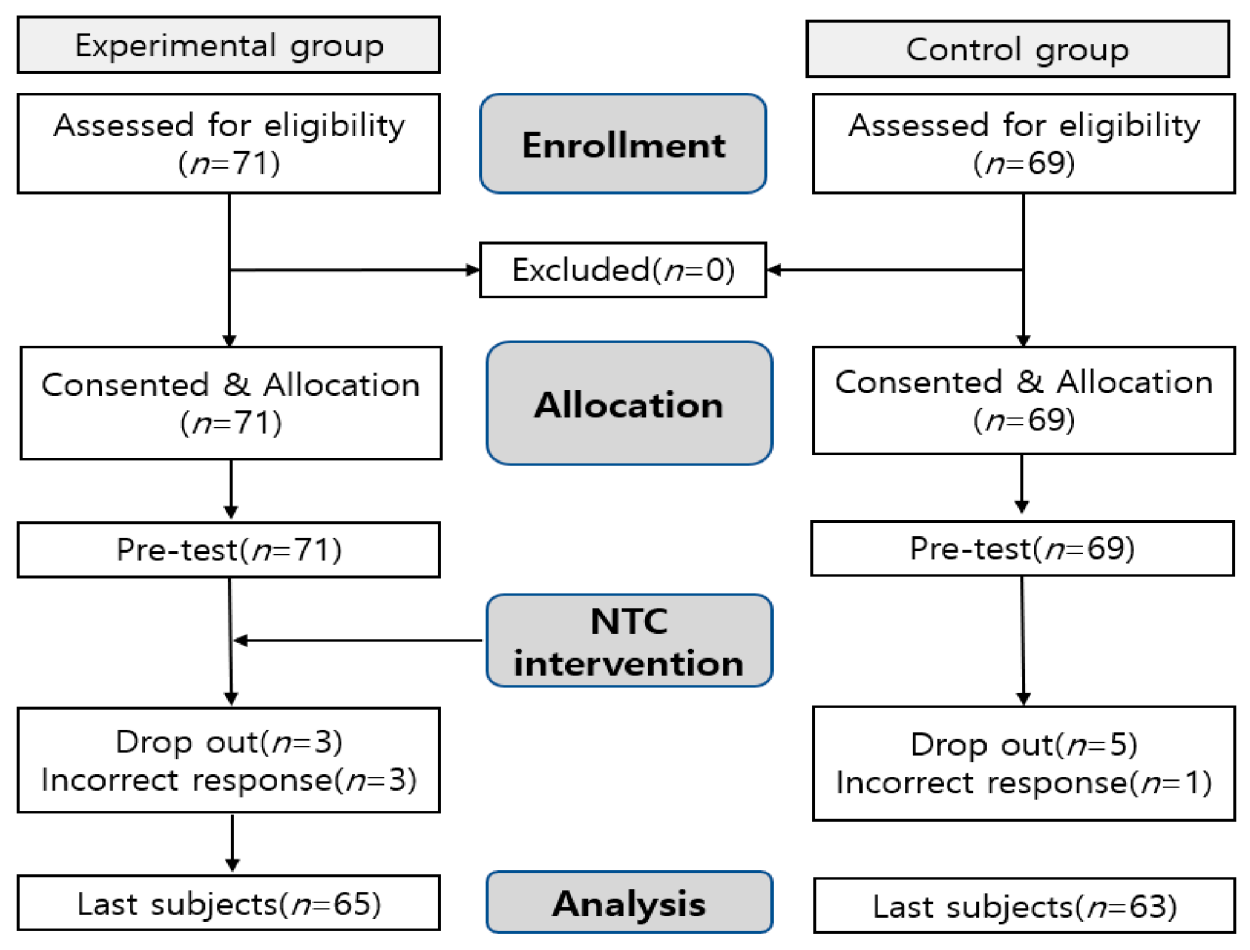
Study flow diagram.

Participants were recruited from an online community primarily comprising parents of infants and toddlers born as high-risk neonates. After the smartphone-based NTC program was introduced, applicants were prioritized for the experimental group and invited to use the app; the control group was recruited after the experimental group was fully enrolled. We recruited 71 participants for the experimental group and 69 for the control group. After excluding dropouts (3 in the experimental group and 5 in the control group) and invalid responses (3 experimental, 1 control), 65 experimental and 63 control participants remained (Figure 2).

### 3.3. Measurements

#### 3.3.1. Developmental and Parenting Knowledge

Developmental and parenting knowledge refers to parents’ understanding of children’s physical, cognitive, and emotional development and appropriate parenting practices that support these domains [16]. It was assessed using the Parent Knowledge Scale for the Development and Parenting of High-Risk Infants developed by Park and Bang [16]. The scale comprises 26 developmental knowledge items and 12 parenting items, with dichotomous responses scored 1 for correct and 0 for incorrect or unknown responses. Higher scores indicate greater knowledge [16]. In this study, the reliability coefficient (KR-20) was 0.93 for developmental knowledge and 0.67 for parenting knowledge.

#### 3.3.2. Maternal Confidence

Maternal confidence refers to a mother’s awareness of her child’s needs, confidence in performing parenting tasks, and satisfaction with her maternal role [17]. It was measured using the Maternal Confidence Questionnaire developed by Parker and Zahr [18] and adapted by Chae [17]. The 14-item instrument uses a 5-point Likert scale, with total scores ranging from 14 to 70. Higher scores indicate greater maternal confidence [17,18]. Cronbach’s α was 0.84 in this study.

#### 3.3.3. Parenting Stress

Parenting stress refers to stress experienced when parenting demands exceed parents’ perceived resources or ability to cope [19]. It was measured using the Parenting Stress Index developed by Abidin [19] and modified by Kim [20]. The 35-item tool uses a 5-point Likert scale, with higher scores indicating greater stress [19,20]. Cronbach’s α was 0.93 in this study.

### 3.4. Data Collection

Data were collected through a self-report online survey, requiring approximately 10 min to complete. Data collection occurred from June 2024 to February 2025, with the experimental group surveyed from June to November 2024 and the control group from December 2024 to February 2025. Groups were recruited sequentially to minimize intervention diffusion and information contamination through interactions in online communities. The experimental group completed the pretest after installing the app and providing informed consent. After participating in the program for 5 to 6 weeks, participants received posttest instructions and completed the posttest questionnaire.

The control group comprised eligible participants who voluntarily consented to participate. They completed pre- and posttests 6 weeks apart using the same online survey method and content as the experimental group. During this period, the NTC program app was temporarily closed to prevent intervention spillover and reopened after data collection. After the posttest, information on the NTC program content and use was provided to minimize educational inequality and encourage voluntary use.

### 3.5. NTC Program

The Analysis, Design, Development, Implementation, and Evaluation instructional design model [21] was used to develop and implement an educational NTC program.

**Analysis:** Educational needs and available resources were assessed through a literature review and interviews with the target group about parenting experiences. Findings indicated that support systems in the Republic of Korea were limited and that participants relied on online communities where inaccurate information could spread without expert guidance. Thus, educational programs addressing these gaps are needed [7,21].

**Design:** The program was designed as a transitional care intervention for infants and toddlers at home after NICU discharge, delivered through a smartphone application suitable for home use.

**Development:** In collaboration with a specialized development company, the researchers developed a user-friendly, functional application for parents. Content was based on key attributes identified through a preliminary literature review and initially developed by two neonatologists, one neonatal nurse specialist, one rehabilitation therapist, and one nursing professor. For content validation, one NICU nursing team manager and two neonatologists reviewed the program. Two parents experienced in raising premature infants pilot-tested the program, leading to refinements in content organization and delivery. The program was finalized after evaluating its algorithm, heuristics, usability, and overall quality and was named “Developing Together.”

The curriculum comprised four sections: parenting diary, education, professional coaching, and peer community. Detailed content is presented in Table 2.

**Implementation:** The experimental group installed and actively used the app on their smartphones. The “parenting diary” enabled participants to record growth data, including height, weight, feeding and elimination patterns, and physical characteristics, and provided reminders for events such as outpatient appointments. The “education” section covered NICU discharge preparation, nutrition, developmental and rehabilitation support, parenting knowledge, medical management, and social support systems, enabling self-directed learning through bulletin boards. The “professional coach” section provided question-and-answer counseling and education from neonatologists, rehabilitation therapists, lactation consultants, and neonatal nurses. Experts responded to inquiries weekly, with one to four interactions per question, depending on content. The “peer community” section included bulletin boards and comments for sharing daily experiences, emotions, children’s growth photos, and parenting knowledge.

The app had no minimum participation requirement, but participants were encouraged to use the educational bulletin board and growth journal at least 5 days per week. Administrators monitored login, bulletin board review, and growth journal entry frequencies to assess engagement. Participants using the app fewer than five times weekly received weekly notifications and text messages encouraging participation (Table 3). The intervention lasted 5 to 6 weeks.

**Evaluation:** An additional survey was conducted during the post-program assessment to evaluate app quality, including the appropriateness of the usage period, convenience, intention to continue use, and willingness to recommend the app. Most participants rated the program positively (Table 3). Feedback was analyzed, and straightforward adjustments were implemented immediately, while broader issues were incorporated into the future development roadmap.

### 3.6. Data Analysis

Data were analyzed using IBM SPSS Statistics v26 (IBM Corp., Armonk, NY, USA). Participant characteristics were summarized using frequencies, percentages, means, and standard deviations. Homogeneity of general characteristics and dependent variables was assessed using *t*-tests, χ^2^ tests, and Fisher exact tests. Program effectiveness was evaluated using ANCOVA. A per-protocol approach was used for the final analysis. Missing data from dropouts or incorrect posttest responses were handled by listwise deletion; thus, only participants who completed the program and all assessments (*N* = 128) were included in the final ANCOVA. Measurement reliability was assessed using the Kuder-Richardson formula 20 and Cronbach’s α coefficient.

### 3.7. Ethics Approval

The study adhered to the Declaration of Helsinki. It was approved by the Institutional Review Board (IRB no. 1041566-202402-HR-004-01) of Chungwoon University. The informed consent form described the study purpose, procedures, participation requirements, risks and benefits, protection of personal information, voluntary withdrawal, and compensation. Before enrollment, participants watched an explanatory video and provided written consent. Data were coded to ensure confidentiality and anonymity. Sensitive data were accessible only to the principal investigator and were not used beyond the study objectives.

## 4. Results

### 4.1. Homogeneity Testing of Participants

No significant between-group differences were found in general characteristics (Table 4). Mean parent age was 34.55 ± 3.31 years in the experimental group and 33.79 ± 5.66 years in the control group (t = 0.93, *p* = 0.354). Most participants were middle class and had a college degree or higher. All primary caregivers were mothers, and most deliveries were by cesarean section. Mean corrected infant age was 7.86 ± 6.85 months in the experimental group and 6.73 ± 6.32 months in the control group (t = 1.54, *p* = 0.125). Mean current infant weight was 6.07 ± 2.51 kg and 6.91 ± 2.45 kg, respectively (t = −1.92, *p* = 0.057). However, the breastfeeding rate, including mixed feeding, was significantly higher in the experimental group than in the control group (58.4% vs. 25.4%; χ^2^ = 14.34, *p* < 0.001).

### 4.2. Effectiveness of the Program

To test the hypotheses, analysis of covariance (ANCOVA) was performed with breastfeeding status as a covariate to control for baseline differences between groups. Before analysis, ANCOVA assumptions were assessed. The group × breastfeeding status interaction was not significant, supporting the homogeneity of regression slopes. The Shapiro–Wilk test indicated normally distributed residuals, and Levene’s test confirmed homogeneity of error variances. Thus, all assumptions were met, and the final ANCOVA model was performed (Table 5).

Hypothesis 1 (Experimental and control groups receiving the smartphone-based NTC program would differ in terms of developmental knowledge) was supported. Developmental knowledge differed significantly between groups, F (3, 123) = 3.95, *p* = 0.009, partial η^2^ = 0.045. After adjustment for breastfeeding status, the posttest mean score was 19.08 (SE = 0.76; 95% CI, 17.58–20.58) in the experimental group and 17.16 (SE = 0.77; 95% CI, 15.64–18.69) in the control group, indicating significantly higher developmental knowledge among participants receiving the intervention.

Hypothesis 2 (Experimental and control groups receiving the smartphone-based NTC program would differ in parenting knowledge) was supported. Parenting knowledge differed significantly between groups, F (3, 123) = 6.72, *p* < 0.001, partial η^2^ = 0.074. After adjustment for breastfeeding status, the posttest mean score was 8.57 (SE = 0.15; 95% CI, 8.27–8.86) in the experimental group and 7.93 (SE = 0.15; 95% CI, 7.63–8.23) in the control group, indicating significantly higher parenting knowledge among participants receiving the intervention.

Hypothesis 3 (Experimental and control groups receiving the smartphone-based NTC program would differ in maternal confidence) was supported. Maternal confidence differed significantly between groups, F (3, 123) = 9.33, *p* < 0.001, partial η^2^ = 0.100. After adjustment for breastfeeding status, the posttest mean score was 56.54 (SE = 0.79; 95% CI, 54.97–58.11) in the experimental group and 51.96 (SE = 0.81; 95% CI, 50.36–53.55) in the control group, indicating higher maternal confidence among participants receiving the intervention.

Hypothesis 4 (Experimental and control groups receiving the smartphone-based NTC program would differ in parenting stress) was supported. Parenting stress differed significantly between groups, F (3, 123) = 2.70, *p* = 0.046, partial η^2^ = 0.031. After adjustment for breastfeeding status, the posttest mean score was 84.70 (SE = 1.97; 95% CI, 80.80–88.59) in the experimental group and 91.11 (SE = 2.01; 95% CI, 87.15–95.07) in the control group, indicating lower parenting stress among participants receiving the intervention.

## 5. Discussion

This study found that the smartphone-based NTC program “Developing Together” improved parental knowledge and maternal confidence and reduced parental stress among families of high-risk infants and toddlers. The study findings and implications are discussed below.

“Developing Together” improved maternal confidence and knowledge and reduced parenting stress during the transition to home care. Improvements in developmental and parenting knowledge (Hypothesis1 and Hypothesis 2) and maternal confidence (Hypothesis 3) were also reported by Phagdol et al. [22], who implemented a home-based mHealth application intervention, and Hägi-Pedersen et al. [23], who provided video-based, face-to-face education. These findings are consistent with the present study. Similarly, the reduction in parenting stress (Hypothesis 4) aligns with Kubicka et al. [9], who reported reduced parental stress and improved parent-staff communication among parents participating in the FICare program.

In particular, the “expert coaching” and “peer community” components addressed limitations of online communities, including misinformation. The program also strengthened emotional support from health care professionals, consistent with Franck et al. [14], who reported positive parental evaluations of clinical rounds, parent group classes, and peer mentoring in a transitional nursing application program. Parenting stress decreased with multidisciplinary expert coaching and parenting guidance, consistent with studies [24,25] showing that nurses’ emotional and educational support reduces parenting stress. These findings suggest that appropriate expert support can strengthen parents’ parenting competencies.

The “parenting diary” enabled recording of growth and development data, including feeding and weight, and provided reminders for important schedules. These features facilitated collection of developmental data from high-risk infants during the transition period and may provide epidemiological indicators of transitional care in the Republic of Korea. Data accumulated through the app on breastfeeding, skin-to-skin contact, parental experiences, and responses may support future community programs and policy development [12,15]. However, as this was a short-term, grant-funded project, long-term sustainability remains challenging, particularly regarding operating costs and connections with medical specialists. Future efforts should establish sustainable operations through collaboration with community health centers and similar institutions.

Domestic NTC initiatives include kangaroo care protocols for parents [26] and discharge programs [27,28]. However, these initiatives have largely been concentrated in large hospitals in major urban centers [11]. E-health intervention can address these geographic and logistical constraints. Mobile apps reduce physical barriers, strengthen interactions between health care providers and parents, and improve accessibility [29], providing an effective means of high-quality care and support for families experiencing isolation during the transition period. Therefore, efforts should continue to develop diverse educational programs using emerging smart-device and artificial intelligence technologies. The “Developing Together” program represents an important step toward establishing NTC in the Republic of Korea. Its smartphone-based format enables flexible application among families in communities outside urban centers. Grounded in FICare theory, the program enabled parents to lead learning and participate in caring for high-risk infants. This approach may provide an academic and practical foundation for implementing FICare in domestic communities and demonstrate its potential for digital transformation.

This section addresses program and research limitations and recommendations for future research. First, the program did not include peer support from experienced FICare parents. Studies [10,13] have shown that parents value peer support from experienced NICU parents during FICare sessions and collective support through shared learning and empathy. Future research should strengthen domestic transitional care nursing programs by incorporating peer-support training components.

Among parenting-related characteristics, breastfeeding method, which may influence parenting experiences and confidence, differed significantly between groups at baseline. ANCOVA was used to adjust for this imbalance, and intervention effects remained statistically significant for the primary outcome after adjustment.

The baseline differences in breastfeeding status may have resulted from the sequential recruitment strategy. To minimize contamination, the experimental group was recruited and received the intervention before the control group. This time-lagged data collection may have introduced temporal variations in breastfeeding practices and other participant characteristics. Although this approach reduced potential intervention contamination, recruiting groups at different times may have affected baseline comparability. Thus, selection and temporal biases cannot be completely excluded. Although ANCOVA adjusted statistically for baseline differences in breastfeeding method, this adjustment cannot fully eliminate biases related to different recruitment periods. Future studies should consider concurrent recruitment with randomization or cluster randomization when contamination is a concern to minimize intervention contamination, selection bias, and temporal effects.

The KR-20 reliability coefficient for Park and Bang’s [16] Parental Knowledge Scale was 0.67 in this study, indicating relatively limited internal consistency despite being marginally acceptable for exploratory research [30]. This lower reliability may reflect differences in participant characteristics. In Park and Bang’s study [16], most infants were 2–3 months old, whereas those in the present study were 1–29 months old, with a mean age of approximately 6–7 months. The inclusion criterion of ≤3 years, based on the catch-up growth trajectory of high-risk neonates, resulted in a highly heterogeneous age range. Consequently, developmental diversity may have reduced measurement reliability. The broad age range and heterogeneous parenting experiences may have decreased response consistency, while differences in infants’ developmental stages and care needs may have affected the relevance of individual knowledge items. Future research should narrow the participant age range to assess stage-specific efficacy or use age-specific tools rather than a single instrument across a broad developmental span. Studies using this instrument should also conduct item-level analyses of difficulty and discrimination to identify items contributing to low internal consistency. Further assessment of the scale’s dimensionality and construct validity using factor analysis may be warranted. Alternatively, a knowledge assessment tool specifically designed for parents of high-risk infants could be developed and validated across developmental stages and clinical characteristics.

This study assessed program effectiveness based on parental outcomes, including parenting knowledge, stress, and confidence, but not infant growth or development. The short intervention period and home-based postdischarge setting limited longitudinal assessment of infant outcomes. Program effectiveness was assessed using parent-reported questionnaires. Although parental perceptions and behaviors are important indicators of efficacy, reliance on self-report may limit comprehensive outcome assessment. Future studies should include child outcomes, such as growth and development, alongside changes in parental knowledge, perceptions, and behaviors. Collaboration with public health institutions and adequate resources will also be needed to support such multidimensional assessments.

Finally, although individual intervention participation and app-use metrics, including login frequency, participation, and growth-recording activities, were monitored, they were not systematically quantified or analyzed because of technical limitations. This unmeasured variation in engagement may have confounded the primary outcomes. Future studies should systematically assess app-use patterns and adherence using automated tracking systems. Such improvements could clarify the dose–response relationship between intervention adherence and educational outcomes while accounting for potential confounding factors.

## 6. Conclusions

This study developed and implemented a smartphone-based NTC program for high-risk infants and toddlers and their families, grounded in FICare theory, and evaluated its effectiveness in improving knowledge and maternal confidence and reducing parenting stress. The program included a parenting diary, educational content, professional coaching, and a peer community. With professional input, the demand-tailored smartphone application supported home-based education and demonstrated its utility for transitional care. The study also provides foundational data for information collection and efficient service delivery to support families of high-risk infants and toddlers during transitional care.

Based on these findings, future research should develop peer-support training programs for domestic clinical settings and conduct longitudinal studies to inform policies that support family adjustment after NICU discharge and during community-based care.

## Figures and Tables

**Table 1:** FICare’s main contents by Franck et al. [12].

Primary Attribute	Contents
Clinical team and alumni parent training	Nurses and alumni parents are trained in the FICare curriculum. Neonatologists, therapists, and social workers receive in-service education specific to their roles.
Parent education	The parent’s role as primary caregiver for the infant, and orientation to the NICU and parent resources
Direct care	Provide as much infant care as possible, with support from nurses (excluding ventilation management, intravenous fluid or intravenous medication administration, etc.)
Daily medical rounds	Receive updates on the infant’s condition, ask questions, and agree with the rounding team on the infant’s daily plan
Peer Support	Receive peer-to-peer support from alumni parents who will contact them remotely
Documentation	Document time spent with their infant and record infant caregiving (e.g., skin-to-skin, feeding), observations, and skills acquisition

**Table 2:** Contents of the application program for families of preterm infants.

Theme	Contents	Methods	Strengths
Parenting Diary	Height and Weight measurement Feeding and Nutrition: feeding, weaning, elimination Physical Examination: special cues, consultation record Alarm: medical follow-up schedule	Parent self-reporting	Integrated and systematic monitoring
Education	Developmental management: development of premature-infants, rehabilitation exercises and play Nutritional Management: Breastfeeding and complementary feeding knowledge Medical Management: Emergency response procedures, key developmental milestones Preterm-specific vaccination knowledge Provision of national support policies and regional assistance information	Board reviews	Providing tailored parenting information Detailed Educational Content Based on Developmental Milestones
Professional Coach	Multidisciplinary treatment team: neonatologist, rehabilitation therapists, lactation consultants, and neonatal nurses Expert counseling and coaching	Coaching system of Q&A type	Providing expert parenting guidelines
Peer Community	Daily life and journal entries Empathy-based peer interaction Internet-based self-help group activities among parents	Interaction by Board and comment	Peer interaction Alleviating social isolation

**Table 3:** Program Participation and Quality Assessment of control group (*n* = 63).

	Contents		Frequency Range (Per Person) or *N* (%)
Program usage monitoring	login frequency weekly		4~21
	bulletin board review frequency weekly		11~27
	growth record frequency weekly		1~6
evaluate the quality of the application	Suitability usage period	Yes	47 (74.6)
		No	10 (15.8)
		Un-response	6 (9.6)
	convenience	Yes	61 (96.8)
		No	2 (3.2)
	intention to continue using	Yes	51 (80.9)
		No	12 (19.1)
	willingness to recommend	Yes	60 (95.2)
		No	3 (4.8)

**Table 4:** Homogeneity of general characteristics between groups (*n* = 128).

Characteristic		Experimental (*n* = 65)	Control (*n* = 63)	t or χ^2^	*p*
		*N* (%) or M ± SD			
Parent	Age (years)	34.55 ± 3.31	33.79 ± 5.66	0.93	0.354
	Education			0.04	0.947
	<College degree	8 (12.3)	8 (12.7)		
	≥College degree	57 (87.7)	55 (87.3)		
	Job			0.27	0.603
	Yes	36 (55.4)	32 (50.8)		
	No	29 (44.6)	31 (49.2)		
	Economic status				
	High	2 (3.2)	5 (7.9)	1.66	0.436
	Middle	55 (84.5)	49 (77.8)		
	Low	8 (12.3)	9 (14.3)		
	Delivery				
	Vaginal	16 (24.6)	11 (17.5)	0.98	0.321
	C/S	49 (75.4)	52 (82.5)		
	Feeding				
	Breast/mixed feeding	38 (58.5)	16 (25.4)	14.34	<0.001
	Formula feeding	27 (41.5)	47 (74.6)		
Infant	Age (week)				
	Gestational (week)	33.18 ± 2.89	32.36 ± 3.15	1.54	0.125
	Corrected (month)	7.86 ± 6.85	6.73 ± 6.32	0.97	0.334
	Weight				
	Birth (kg)	2.05 ± 0.58	1.96 ± 0.70	0.847	0.398
	Current (kg)	6.07 ± 2.51	6.91 ± 2.45	−1.92	0.057
	Birth order				
	First	58 (89.2)	49 (77.8)	4.04	0.132
	Second	7 (10.8)	12 (19.0)		
	Third or more	0 (0)	2 (3.2)		
	Twin				
	Yes	3 (4.6)	8 (12.7)	0.12 ^†^	0.093
	No	62 (95.4)	55 (87.3)		
	Illness on NICU				
	Yes	40 (61.5)	32 (50.8)	1.50	0.221
	No	25 (38.5)	31 (49.2)		

^†^ Fisher’s exact test; C/S, cesarean section.

**Table 5:** Differences of study variables between experimental (*n* = 65) and control groups (*n* = 63).

Variable		Pre-Test	Post-Test	Adjusted Post-Test Mean (SE)	95% CI	F (*p*)	Partial η^2^
		M ± SD					
Development knowledge	Exp.	15.67 ± 7.68	19.16 ± 5.81	19.08 (0.76)	17.58–20.58	3.94 (0.009)	0.045
	Cont.	16.31 ± 5.84	17.07 ± 4.22	17.16 (0.77)	15.64–18.69		
Parenting knowledge	Exp.	7.70 ± 1.30	8.55 ± 1.18	8.57 (0.15)	8.27–8.86	6.72 (<0.001)	0.074
	Cont.	7.79 ± 1.20	7.95 ± 1.06	7.93 (0.15)	7.63–8.23		
Maternal confidence	Exp.	52.15 ± 4.56	56.33 ± 5.95	56.54 (0.79)	54.97–58.11	9.33 (<0.001)	0.100
	Cont.	51.11 ± 7.45	52.17 ± 7.09	51.96 (0.81)	50.36–53.55		
Parenting stress	Exp.	91.46 ± 12.63	84.26 ± 15.32	84.70 (1.97)	80.80–88.59	2.70 (0.046)	0.031
	Cont.	90.11 ± 18.03	89.32 ± 15.91	91.11 (2.01)	87.15–95.07		

M, mean; SD standard deviation; SE, standard error; CI, confidence interval; Exp, experimental group; Cont, control group.

## Data Availability

The raw data of this study cannot be publicly shared, as required by the Institutional Review Board of Chungwoon University to protect participants’ privacy. However, anonymized summary data is available upon reasonable request to the corresponding author.

## Abbreviations

The following abbreviations are used in this manuscript:

FCC	Family-Centered Care
FICare	Family-Integrated Care
NICU	Neonatal Intensive Care Unit
NTC	Neonatal Transitional Care
